# New Acidic Precursor and Acetone-Based Solvent for Fast Perovskite Processing via Proton-Exchange Reaction with Methylamine

**DOI:** 10.3390/molecules25081856

**Published:** 2020-04-17

**Authors:** Sergey A. Fateev, Ekaterina I. Marchenko, Andrey A. Petrov, Eugene A. Goodilin, Alexey B. Tarasov

**Affiliations:** 1 Laboratory of New Materials for Solar Energetics, Department of Materials Science, Lomonosov Moscow State University, 1 Lenin Hills, 119991 Moscow, Russia; 2 Department of Chemistry, Lomonosov Moscow State University, 1 Lenin Hills, 119991 Moscow, Russia

**Keywords:** thin films, methylamine, hybrid perovskites, lead halide perovskites, perovskite solar cells, lead precursor, chemical conversion, solution deposition, fast processing, HPbI_3_

## Abstract

A new solvent system for PbI_2_ based on HI solution in acetone with a low boiling point is proposed. High solubility of PbI_2_ is caused by the formation of iodoplumbate complexes, and reaches a concentration of 1.6 M. Upon its crystallization metastable solvate phases PbI_2_∙HI∙n{(CH_3_)_2_CO} are formed. The latter allows for their easy deposition on substrates in a form of smooth and uniform thin films by spin-coating. Through a fast acid-base reaction with a gaseous amine, the films of the intermediate phase can be completely converted to single-phase perovskite films. The developed method allows one to form smooth perovskite films with high crystallinity with a thickness up to 1 μm. Due to easy and fast processing, the developed method can be promising for perovskite technology upscaling.

## 1. Introduction

Arising less than 10 years ago, perovskite photovoltaics have become the fastest growing field of state-of-the-art solar energetics and attract today great attention of the scientific community and industry. The highest power conversion efficiency of PSCs has reached over 25% [1] and the efficiency of devices with an area of more than 100 cm^2^ exceeded 15% [2], which is comparable with the best commercial polycrystalline silicon solar cells. Nevertheless, the main trump card of perovskite solar cells has a presumable low cost of production achieved primarily through an application of simple and cheap solution deposition methods of lead halide perovskites APbX_3_ (where A = CH_3_NH_3_^+^, CH(NH_2_)^+^ or Cs^+^; X = I^−^, Br^−^, Cl^−^) as a light-absorbing layer.

A great variety of solution methods is based on simple reactions occurring upon deposition of APbX_3_ from solutions or perovskite formation from two single salts (AX + PbX_2_), which have a small driving force (ΔG_r_ ≈ 0) [3,4] and a quite low reaction rate [5]. An alternative approach is perovskite formation via proton-exchange reactions (PER) having advantage of fast and highly exothermic (ΔG_r_ < 0) origin. The PER approach based on a chemical conversion of a solid lead-containing precursor (B^1^H)PbI_3_ with a cation (B^1^H^+^) into an organo-inorganic perovskite (B^2^H)PbX_3_ containing another cation (B^2^H^+^) formed by the proton exchange of B^1^H^+^ with an organic base B^2^. The higher the Brønsted–Lowry acidity of the (B^1^H^+^) cation and simultaneously the lower the acidity of the (B^2^H^+^), the greater the driving force of the reaction and the faster the conversion. Following this logic, several authors, starting from Wang [6], have proposed an acidic precursor “HPbI_3_” for the synthesis of CH_3_NH_3_PbI_3_ perovskite via conversion by methylamine vapors. Later, other compounds similar to “HPbI_3_”, such as PbI_2_∙xHI [7,8] and “HPbI_3_∙DMF intermediate” [9], were claimed as suitable acidic precursors for such a synthesis of high quality CH_3_NH_3_PbI_3_ films and further fabrication of highly effective PSCs.

Importantly, in all the reported methods the acidic precursor was obtained from concentrated solutions of PbI_2_ in dimethylformamide (DMF) with aqueous HI [6,7,8,9,10], in which rapid hydrolysis of DMF inevitably takes place [10,11]: 

HCON(CH_3_)_2_ + H^+^ + H_2_O → (CH_3_)_2_NH_2_^+^ + HCOOH.

In the presence of dissolved PbI_2_ and excessive iodide anions, the main product of this reaction, dimethylammonium (DMA^+^), forms a solid phase DMAPbI_3_, which was previously mistaken for the mythical “HPbI_3_” [12,13]. Forming non-perovskite phase, the DMA affects the properties of the resulting perovskite film [11,13,14] (for more detailed discussion see SI). However, even a more significant problem of using DMF-based acid solutions is almost uncontrollable changing of solution composition upon preparation, storage, and processing. Therefore, new solution systems based on solvents with much better stability in the presence of concentrated aqueous HI are required for successful implementation of the PER approach to achieve better reproducibility and reliability of the formation of perovskite films.

Herein, we developed a new binary solvent system acetone—HI_(aq.)_ with a low boiling point and viscosity for deposition of a new highly reactive acidic precursor PbI_2_∙HI∙n{(CH_3_)_2_CO} being highly useful for perovskite production.

## 2. Results and Discussion

### 2.1. Solvation and Crystallization from Acetone–HI_(aq.)_ System

Strong donor solvents such as DMSO and DMF are known to dissolve PbI_2_ at high enough concentration (up to 1.2 M and 0.8 M respectively) due to the formation of donor–acceptor complexes. Although polar solvents with weak donor properties such as acetone dissolve no lead iodide, an addition of iodide anions leads to the formation of iodoplumbate complexes (e.g., [PbI_3_]^−^ and [PbI_4_]^2−^) solvated via strong ion-dipole interaction with solvent molecules. Indeed, we found that the acetone-HI_(aq.)_ system demonstrates surprisingly high solubility of PbI_2_ up to 1.6 M (for the case of HI:PbI_2_ = 1:1; see Figure S1).

**Figure 1:**
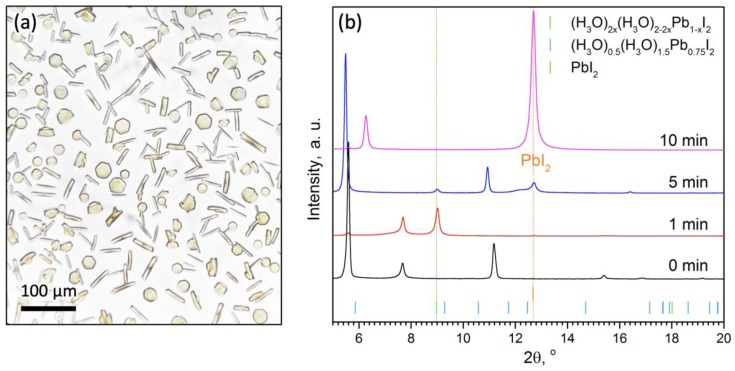
(**a**) Appearance of the crystals of the Phase-1 grown in drop of solution; (**b**) Evolution of the film deposited from 0.375 M solution of HI:PbI_2_ = 1:1 in acetone. Positions of the reflections of the known phases (PbI_2_, (H_3_O)_0.5_(H_2_O)_1.5_Pb_0.75_I_2_), and (H_3_O)_2x_(H_2_O)_2–2x_Pb_1−x_I_2_ (x ≈ 0.23)) are indicated by colored bars.

Acetone solutions have several beneficial features such as low viscosity, high wetting ability, and high volatility, which facilitate the obtaining of smooth and uniform thin films using spin-coating or other solution methods [15]. Due to the rapid evaporation of acetone, the solution of PbI_2_ in acetone-HI_(aq.)_, either in the form of a distributed film or large droplets, quickly concentrates initiating the crystallization. Firstly, transparent crystals with a hexagonal or rod-like shape appear in the drying drops (Figure 1a). The diffraction pattern of the crystals matches with none of the reported phases, which hypothetically form in the presence of the HI aqueous solution (Figure 1b) and therefore represent a new phase denoted hereinafter as the Phase-1. The different shape of the observed crystals may indicate that there are at least two different phases. Alternatively, the observed crystals can belong to one phase while their various shape is governed by different orientation of the seed crystals and their preferential growth in the direction parallel to the plane of the substrate. When separated from solution, the crystals of the Phase-1 decompose in a few minutes with their color turning to yellow, and then transforming into a shapeless mash with a diffraction pattern corresponding to PbI_2_ (Figure 1b, the magenta curve).

### 2.2. Characterization of the Phase-1

**Figure 2:**
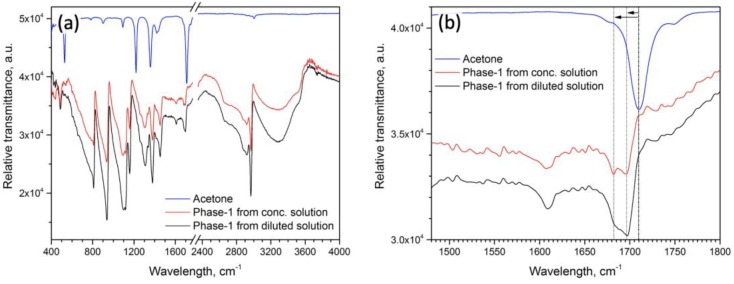
IR-spectrum of the Phase-1 crystals crystallized from saturated (red line) and diluted (black line) solutions in comparison with pure acetone (blue line): (**a**) full spectra and (**b**) region of C=O group stretching vibration—the position in pure acetone indicated by black dashed line and the shifted and split C=O vibration in Phase-1 indicated by dark-grey lines.

The very rapid decomposition of Phase-1 makes it almost impossible to collect single-crystalline diffraction data with sufficient quality to solve the structure, however, a number of indirect data suggests that the Phase 1 is an iodoplumbate with protonated acetone molecule as a cation. The IR spectroscopy data recorded from the Phase-1 fine powder in the mode of attenuated total reflection confirm not only the presence of acetone in the structure, but also indicate a red shift of the carbonyl group vibration (Figure 2a,b). The shift of C=O group stretching vibration frequency toward lower wavenumbers compared to pure acetone (Δν = 13–23 cm^−1^) is typical for oxygen bonding with a proton or a bound proton. 

A careful examination of the diffraction pattern of the Phase-1 powder allows one to notice a two distinct group of reflections corresponding to two independent groups of planes: the first selected group includes the reflections at 5.63°, 11.25° (2θ), and the second group includes the reflections at 7.73°, 15.42°, and 23.26° (2θ; Figure S2). The presence of two strong independent reflections in the small angle region and the corresponding series of distant reflections is a characteristic sign of structures with chain-like motif, which are very common for iodoplumbates with bulk flattened cations (formamidinium [16], dimethylammonium, protonated urea, and protonated amides [11]). Therefore, we proposed a similar structure with a single chains of octahedra connected along the faces for the Phase-1 (Figure S5). In the proposed model structure, the first and second group of reflections corresponded to the h00 and hk0 planes (Figures S3 and S4, Tables S1 and S2), respectively. According to the requirement of charge balance, the structure should also contained one cation for each fragment of the [PbI_3_^−^]_n_ polymer chain (Figure S5), so the most plausible formula of the Phase-1 could be {n(CH_3_)_2_CO∙H^+^}[PbI_3_^−^]. The protonated acetone is expected to be strong and unstable Brønsted–Lowry acid, which explains the above-mentioned strong tendency of the Phase-1 be quickly decomposed with releasing gaseous acetone and HI (Figure S2). On the other hand, the HI is added to acetone in the form of an aqueous solution (57% HI_aq._ has molar ratio H_2_O/HI = 5.6), some of the “hydrated acid” can evaporate much more slowly and remain in the film. Most likely, this explains the formation of such intermediates as (H_3_O)_2x_(H_2_O)_2−2x_Pb_1−x_I_2_ (x ≈ 0.23), which can be seen by XRD (Figure 1b see diffraction pattern after 1 min).

In addition, similarly to other chain iodoplumbates with a high band gap, the Phase-1 also demonstrates very low absorption in the visible region (see Figure S1a), which is an indirect sign of the low dimensionality of the chained inorganic framework.

It should be noted that in accordance with the chemical properties of acetone in the presence of HI as a strong acid, aldol condensation could occur, the main product of which will be mesityl oxide (in the case of concentrated acid). However, unlike the hydrolytic reactions in DMF-HI_(aq.)_ solutions, the product of this possible side reaction cannot form stable cations that can enter the perovskite structure, and moreover mesityl oxide is quite volatile.

### 2.3. Perovskite Films Processing

Expectedly, behaving as an acidic precursor, the Phase-1 reacts vigorously with methylamine forming perovskite:

{n(CH_3_)_2_CO∙H^+^}[PbI_3_^-^] + CH_3_NH_2_ = CH_3_NH_3_PbI_3_ + n(CH_3_)_2_CO↑

**Figure 3:**
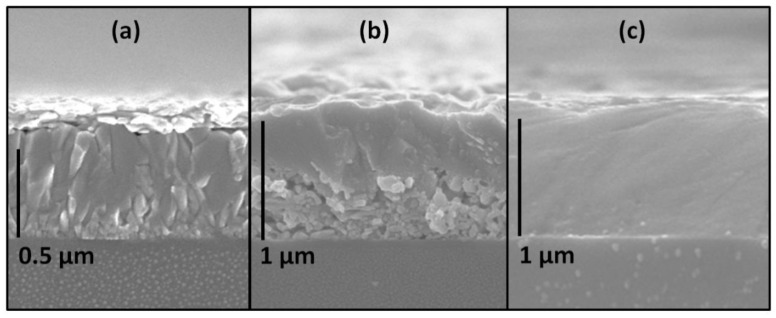
SEM micrographs of the cross-sections of films: (**a**) the precursor film deposited from 0.75 M solution, (**b**) the same precursor film at initial stage of the conversion, and (**c**) the resulting perovskite film after full conversion (20 s) and short annealing (5 min, 100 °C).

**Figure 4:**
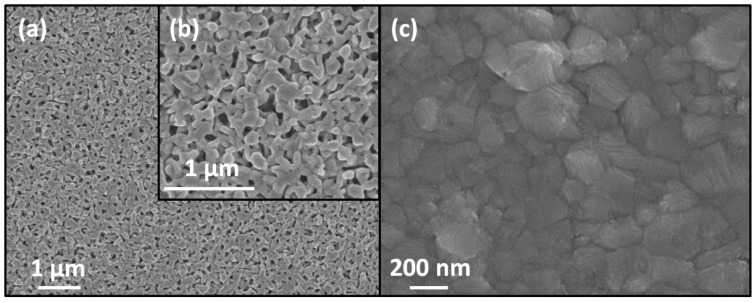
SEM micrographs of top surface of films: (**a**) the precursor film and (**b**) its enlarged fragment, and (**c**) the final perovskite film.

Consequently, the Phase-1 in the form of a thin film can be easily converted to a single-phase perovskite film by either gaseous methylamine or highly diluted solution of methylamine in an appropriate neutral solvent followed by annealing at 100 °C. The precursor film of the Phase-1 was obtained by conventional spin-coating with dropping of the solution in a dynamic mode. High wetting ability and low viscosity ensure uniform spreading of the solution into a thin layer over the entire surface of the substrate. The rapid evaporation of the solvent leads to the instantaneous formation of a large number of nucleation centers resulting in a small-crystalline smooth film with a low roughness (Figure 3a, Figure 4a). The morphology of the adduct films demonstrates a significant number of small pores on the surface that facilitate the penetration of methylamine to the lower boundary of the film, which is favorable for fast and complete conversion (Figure 4a,b).

**Figure 5:**
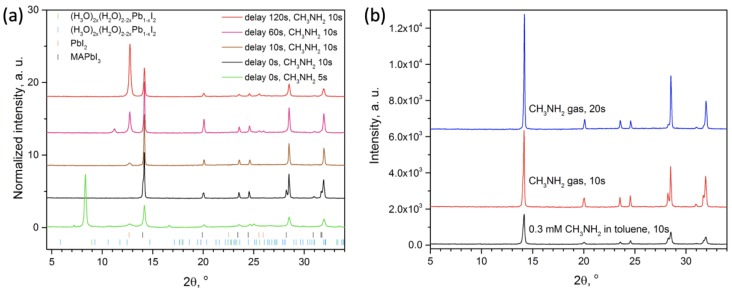
Diffraction patterns (**a**) of the precursor films after different methylamine vapors exposure time (5 and 10 s) and with various delay after deposition (0–120 s) and (**b**) single-phase perovskite films obtaining under conversion by methylamine gas (5 and 10 s of exposure) or diluted methylamine solution in toluene. Positions of the reflections of the known phases are indicated by colored sticks.

According to XRD data, the minimum time required to complete the process of conversion was 8–10 s, processing for a longer time (up to 1 min) did not lead to the formation of impurity phases (Figure 5b). The most important condition for obtaining the final single-phase perovskite film was the conversion of the fresh precursor film immediately after spin-coating (Figure 5a). Even in the case of delay of 1–2 min, the film of the initial precursor ({n(CH_3_)_2_CO∙H^+^}[PbI_3_^−^]) started to decompose with the formation of Pb-enriched phases ((H_3_O)_0.5_(H_2_O)_1.5_Pb_0.75_I_2_) and (H_3_O)_2x_(H_2_O)_2−2x_Pb_1−x_I_2_ (x ≈ 0.23)), see Figure 1b, Figure 5a), which resulted in PbI_2_ impurity in the perovskite films. An excess of CH_2_NH_2_ not only affords full conversion through acid–base reaction but also permits recrystallization resulting in the film with a “monolithic” morphology without observable grain boundaries on the cross-section (Figure 3c), resembling the common appearance of the films after methylamine-induced recrystallization [17].

## 3. Materials and Methods

### 3.1. Materials

Methylammonium iodide (CH_3_NH_3_I), lead iodide (PbI_2_, 99%,) were commercially purchased Dyesol and Sigma-Aldrich respectively and used without further purification. Solutions with a given concentration of HI_(aq.)_ were prepared using concentrated (57 wt%) aqueous iodic acid of *Pro analysis* grade without any stabilizing reducing additives (to avoid oxidation, it was stored under hydrogen in a refrigerator at 0 °C) and acetone of *Purissimum speciale* grade. All solutions were prepared under ambient conditions at 35% humidity, PbI_2_ in different amounts was dissolved in a required volume of mixed solvent (acetone and HI_(aq.)_) and then stirred at room temperature for 5 min until completely dissolved.

### 3.2. Methods

Intermediate solvate crystals growth. Small droplet of a solution was drop-casted onto a cleaned glass slide. After 10–30 s of solvent evaporation, thin films of the evaporating solution were analyzed by an optical microscopy.

Films preparation. Glass substrates were cleaned with detergent, flushed with distilled water and then sequentially washed in ultrasonic baths in acetone, isopropyl alcohol and distilled water. Substrates were further cleaned with UV ozone for 15 min prior to their use.

The precursor films of Phase-1 were obtained by spin-coating of freshly prepared 0.5 M solution of PbI_2_ in acetone-HI_(aq.)_ (HI:PbI_2_ = 1:1) on the clean glass substrates at 6000 rpm for 10 s. It is enough to drop just two drops of the solution (10 μL) onto a rotating glass substrate of 1 cm^2^, the solvent instantly evaporates and a translucent pale yellow film of the precursor forms. Then the precursor films immediately transferred into Petri dish filled with methylamine vapors produced over saturated 35% CH_3_NH_2_ isopropanol solution. Alternatively, conversion of the Phase-1 films into perovskite was conducted in the liquid media of 0.3 M solution of CH_3_NH_2_ in toluene. In both cases, blackening of the film took place on average in 2 s, and subsequent discoloration in 8–10 s. Then the film was transferred to a plate heated to 100 °C and annealed for 2–5 min to remove residual methylamine. In this case, only highly volatile solvents such as acetone, HI and methylamine are involved in the process; therefore, a longer annealing (30 min), which is usual for traditional solution methods of perovskite films fabrication, is not necessary.

In addition, it was shown that the stage of chemical conversion could be combined with the stage of spin-coating by blowing the methylamine vapor onto the film immediately after dropping of the solution and additional 5 s of rotation. In this case, the duration of the entire preannealing stage is not more than 20 s.

### 3.3. Characterization

The XRD measurements were performed with a Bruker D8 Advance diffractometer (CuKα, λ = 1.5406 Å) in the range of 2θ = 3–35° with 0.02° step and 0.1 s step time for perovskite films. The morphology of the films was investigated using Carl Zeiss NVision 40 field-emission scanning electron microscope with EDX detector (Oxford instruments). For cross-section observations, the samples were coated with an ultrathin (<10 nm) layer of Cr to avoid sample charging. The IR spectra of the as-grown Phase-1 crystals were recorded in the attenuated total reflection mode in spectral range 400–4000 cm^−1^ with a resolution of 4 cm^−1^ on IR Fourier spectrometer Perkin Elmer Spectrum 65. For each spectrum as well as for the background, 128 scans were averaged.

## 4. Conclusions

To sum up, we firstly proposed a new mixed solvent system, acetone–HI_(aq.)_, free from uncontrolled hydrolysis and demonstrating high solubility of PbI_2_ up to 1.6 M. The solution of PbI_2_ acetone–HI_(aq.)_ mixture gives an unknown iodoplumbate phase upon crystallization. Based on the IR spectroscopy and XRD data, we found that the discovered phase contains protonated acetone and suggested that it has an inorganic sublattice with one-dimensional chain of [PbI_3_]_∞_ face-sharing octahedral and a general formula {n(CH_3_)_2_CO∙H^+^}[PbI_3_^−^]. Being a metastable acidic precursor, this phase readily reacts with methylamine to form CH_3_NH_3_PbI_3_ perovskite via fast proton-exchange reaction. The developed method allows one to obtain smooth and uniform perovskite films in extremely short time (ca. 5 min from deposition of the precursor film to the end of annealing). Due to easy and fast processing, the developed method can be promising for upscaling and reducing the cost of PSCs production.

## Supplementary Materials

The file of supplementary information is available online, and containing the following information: Figure S1: Optical microscopy photos of the crystals of the adduct: freshly grown (a), aged in 1 min (b), aged in 2 min (c); Figure S2: Diffraction pattern of the Phase-1 powder. The captions indicate the positions of reflections, symbols “x” and “y” indicate the affiliation for one of the two distinct groups of reflections; Table S1: The best fitted refined lattice parameters of the Phase-1 within assumption of orthorhombic unit cell; Figure S3: Diffraction pattern of the Phase-1 in *P**bcm* space group (parameters listed in Table S2): experimental (black) and simulated (red); Table S2: The best fitted refined lattice parameters of the Phase-1 within assumption of monoclinic unit cell; Figure S4: Diffraction pattern of the Phase-1: with *P*2_1_/*c* space group (parameters listed in Table S2): experimental (black) and simulated (red); Figure S5: Probable crystal structure of Phase-1 (space group *Pbcm*) with a single chains of PbI_6_ octahedra in yz (a) and xy (b) projections.
