# Supplementary material for: New Acidic Precursor and Acetone-Based Solvent for Fast Perovskite Processing via Proton-Exchange Reaction with Methylamine
Source: Molecules. 2020 Apr 17;25(8):1856. doi: 10.3390/molecules25081856 (PMC7221818; doi:10.3390/molecules25081856)
Supplement: Supplementary file 1 [file molecules-25-01856-s001.pdf]

Supplementary Materials

New acidic precursor and acetone-based solvent for fast perovskite processing via proton-exchange reaction with methylamine

Sergey A. Fateev[1], Ekaterina I. Marchenko[1], Andrey A. Petrov[1], Eugene A. Goodilin[1,2], Alexey B. Tarasov[1,2]*

[1] *Laboratory of New Materials for Solar Energetics, Department of Materials Science, Lomonosov Moscow State University; 1 Lenin Hills, 119991, Moscow, Russia*

[2] *Department of Chemistry, Lomonosov Moscow State University; 1 Lenin Hills, 119991, Moscow, Russia*

* *Correspondence: alexey.bor.tarasov@yandex.ru (A.B.T.)*

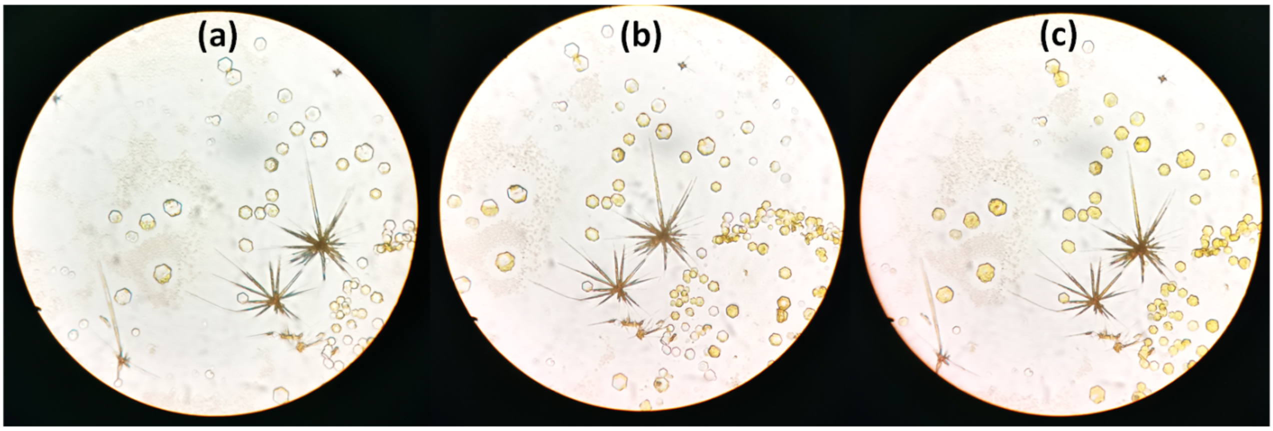

Figure S1. Optical microscopy photos of the crystals of the adduct: freshly grown (a), aged in 1 minute (b), aged in 2 minutes (c).

Assumptions about the crystal structure of Phase-1 and refinement.

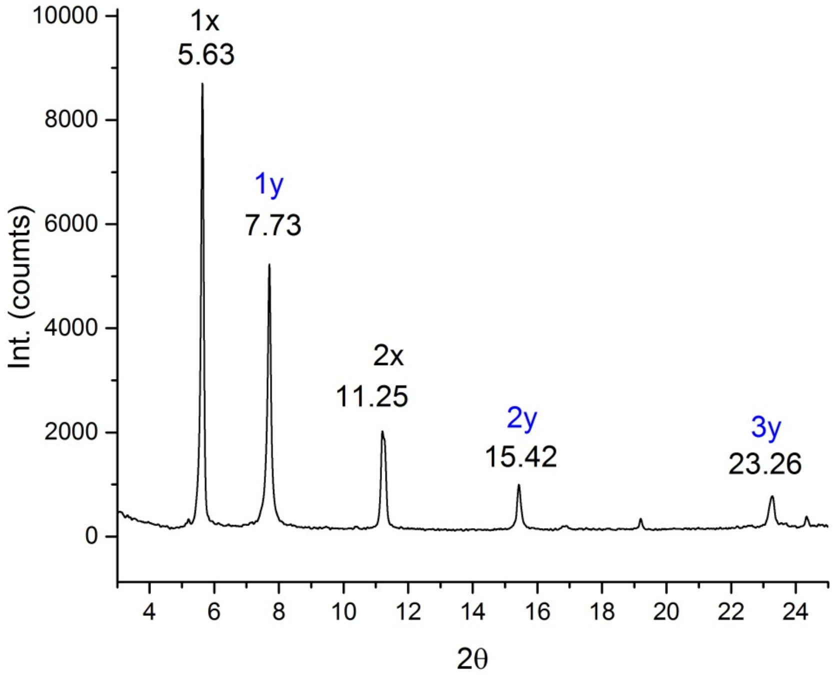

Figure S2. Diffraction pattern of the Phase-1 powder. The captions indicate the positions of reflections, symbols “x” and “y” indicate the affiliation for one of the two distinct groups of reflections.

Assuming the structure with a single chains of octahedrons connected along the faces for the Phase-1, we consider two possible variants of unit cells and chain location: the first for the case of orthorhombic unit cell with chain orientation along c axis and the second one for the case of monoclinic unit cell (β > 90) with the same chain orientation.

Table S1. The best fitted refined lattice parameters of the Phase-1 within assumption of orthorhombic unit cell.

**Space group**	*Pbcm* (№57)
**a, Å**	15.809(1)
**b, Å**	16.832(5)
**c, Å**	7.8732(9)
**α, °**	90
**β, °**	90
**γ, °**	90
	GOF = 2.64<br>Rp = 9.36<br>wRp = 13.34

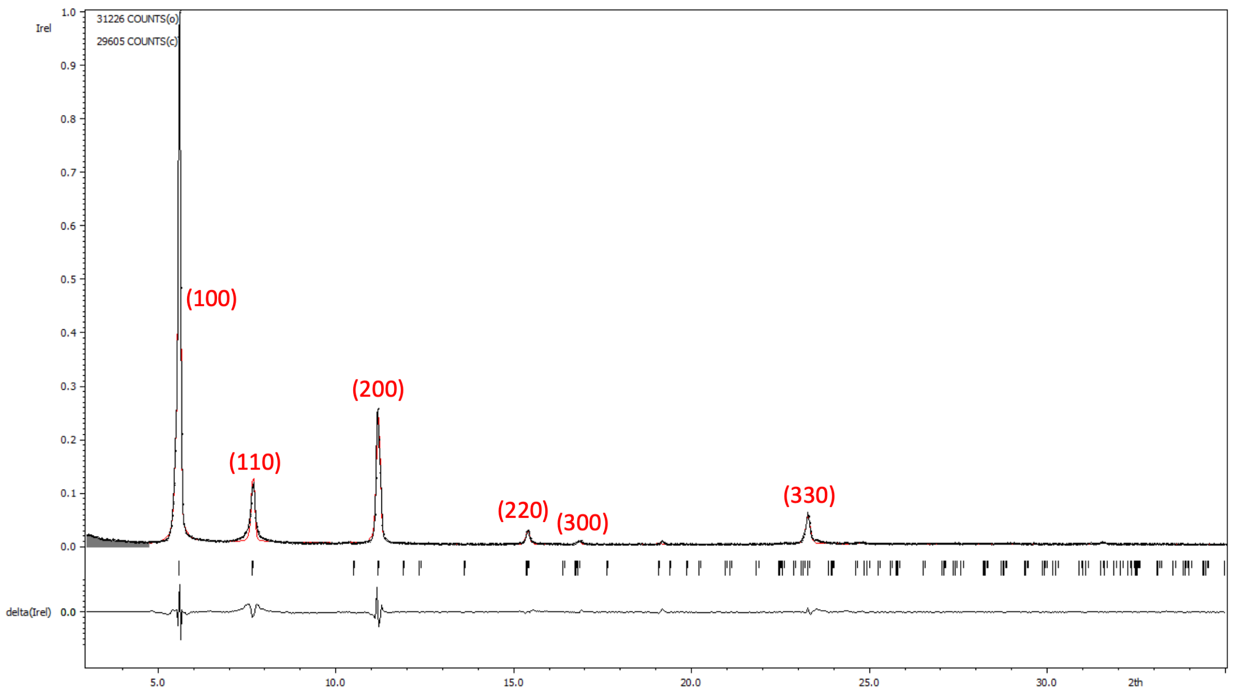

Figure S3. Diffraction pattern of the Phase-1 in *P*bcm space group (parameters listed in Table S2): experimental (black) and simulated (red).

Table S2. The best fitted refined lattice parameters of the Phase-1 within assumption of monoclinic unit cell.

**Space group**	*P*21/*c* (№14)
**a, Å**	16.252(8)
**b, Å**	16.83(1)
**c, Å**	8.14(1)
**α, °**	90
**β, °**	103.4(1)
**γ, °**	90
	GOF = 2.74<br>Rp = 9.99<br>wRp = 13.83

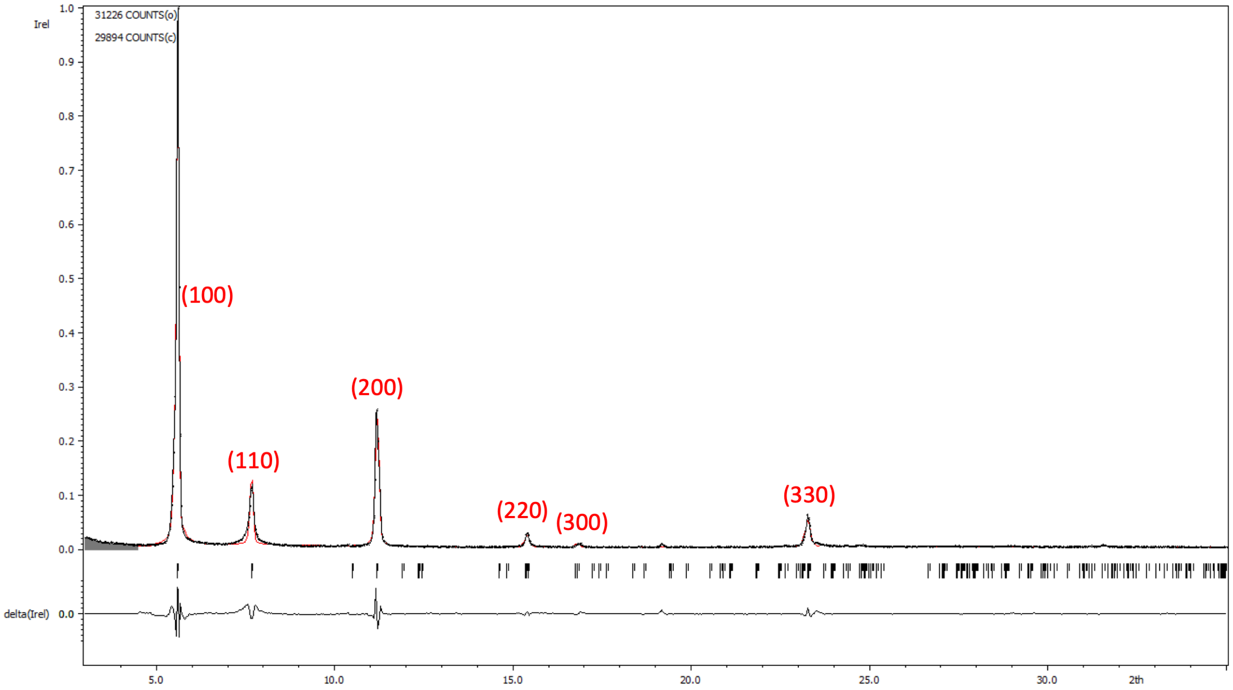

Figure S4. Diffraction pattern of the Phase-1: with *P*21/*c* space group (parameters listed in Table S2): experimental (black) and simulated (red).

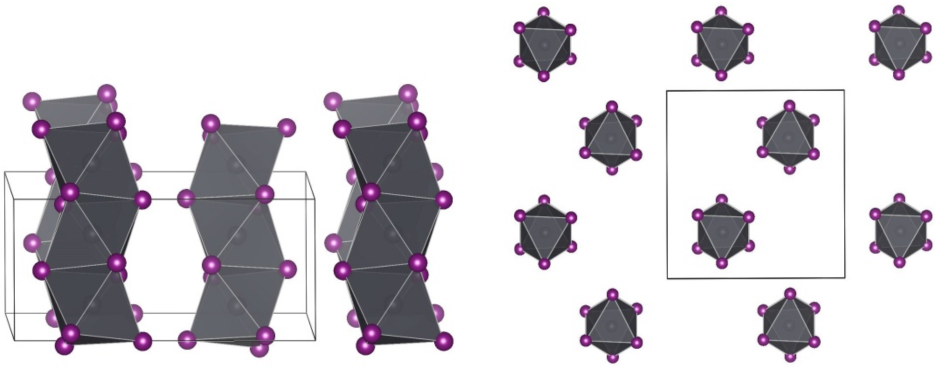

(a) *(b)*

Figure S5. Probable crystal structure of Phase-1 (space group *Pbcm*) with a single chains of PbI_6 octahedra in *yz* (a) and *xy* (b) projections.
